# Sulfur Mustard Effects on Mental Health and Quality-of-Life: A Review

**Published:** 2014

**Authors:** Seyed Mansour Razavi, Zahra Negahban, Mohsen Pirhosseinloo, Mahdiyeh Sadat Razavi, Gholamreza Hadjati, Payman Salamati

**Affiliations:** 1 Professor, Department of Community Medicine, School of Medicine, Tehran University of Medical Sciences, Tehran, Iran.; 2 Department of Health Services Management, School of Public Health, Tehran University of Medical Sciences, Tehran, Iran.; 3 Department of Cognitive Sciences, Institute for Cognitive Science Studies, Shahid Beheshti University, Tehran, Iran.; 4 Assistant Professor, Department of Psychiatry and Psychology, Aerospace Medical School, Artesh University of Medical Sciences, Tehran, Iran.; 5 Professor, Department of Violence Prevention, Sina Trauma and Surgery Research Center, Tehran University of Medical Sciences, Tehran, Iran

**Keywords:** Chemical Warfare, Iran, Mental Health, Mustard Gas

## Abstract

**Objective:** Mental disorders are more common among the chemically injured veterans rather the than the normal population. The main aim of this study was to evaluate the effects of mustard gas (MG) on mental health and quality-of-life (QOL) in the people exposed to it based on reviewing valid published articles.

**Methods:** We searched English databases including Medline, ISI and Scopus as well as Farsi databases including Iranmedex and Irandoc and reviewed them. The used keywords were in two English and Farsi languages. Forty related full texts out of more than 300 articles were assessed and for their qualification, only the publications in accredited journals were considered sufficient.

**Results: **The average mental health score of victims using the general health questionnaire (GHQ) was 48.92. The frequency of anxiety was (18-65%), insomnia (13.63%), social performance disturbances (10.73%), severe depression (6-46%), low concentration (54%), emotional problems (98%), behavioral abnormalities (80%), thought processing disturbances (14%), memory impairment (80%), personality disorders (31%), seizures (6%), psychosis (3%). Post-traumatic stress disorder (PTSD) is one of the most common and important disorders with lifetime PTSD (8-59%), current PTSD (2-33%) and the QOL in chemical warfare victims decreased.

**Conclusion:** Exposure to chemical weapons may lead to physical, mental, social, and economic damages and consequently decrease the victims’ (QOL. Therefore, they should be taken into more care.

## Introduction

According to World Health Organization definition, health is a state of physically, mentally, socially (and spiritually) well-being, not just the absence of disease or disability. The concept of mental health includes subjective well-being, feeling self-empowerment, autonomy, competence, understanding the others and the ability to distinguish his potential intellectual and emotional capabilities. In the other words, a person who is mentally healthy, is capable of coping with his life with the usual stress, he is a useful and productive person in the society and cooperating with others (1). War is a phenomenon that in addition to affecting human biology, it may lead to deep and prolonged complications on their psychological status.

Psychological symptoms in wars have long been considered. For example, during the civil wars in America, the symptoms caused by the war memories were called “soldier’s heart syndrome” (2). Furthermore, during the US-IRAQ war, similar symptoms appeared that it was called the Persian Gulf syndrome (3). During the 8-year war (1980-1988), about 44 000 Iranian soldiers lost their lives during the 242 chemical attacks (4).

Numerous studies performed by Iranian researchers showed that this agent could lead to a wide variety of early and late complications including respiratory (5-6), ocular (7), dermatological (8), immunohematological (9), psychological disorders and other toxic effects in exposed people. The late effects can continue even 40 years later after the initial exposure (10).

Although chemical weapons lead to many physical complications on victims, psychological events are more critical (11(. Psychological consequences will lead to the decrease of l quality-of-life (QOL) (12) and may even continue for two generations. Therefore, identifying and preventing them are important (13). Vedder among the World War I victims and Balali-Mood among the Iraq-Iran war veterans reported mood, anxiety and post-traumatic stress disorder (PTSD) disorders (14, 15). Mohammadi and Nouri studied 70 Iranian CIV in 1993. They found many disorders including anxiety, major depression, between PTSD and somatization among the patients (16). In addition, researchers reported clinical findings such as weakness, sensory hypersensitivity decreased libido, impotence, poor concentration, neurological and cardiac rhythmic disorders among German workers exposed to mustard gas (MG) (17, 18). Because there were not such heavy chemical attacks against any countries in the world along the past decades, also, due to the specific cultural conditions in our country, we could not use the experiences of the other countries and we must study the current situation of our country ourselves (19).

Therefore, the main aim of this study was to evaluate the effects of MG on producing mental health disturbances like anxiety disorders (especially PTSD), personality disorders, behavioral problems, somatization disorder, sleep disturbances, impaired thinking and memory processes, abnormal responses to stress, psychotic and mood disorders as well as QOL for those exposed to MG based on reviewing the valid published articles.

## Materials and Methods

We searched English databases including Medline, ISI and Scopus and Iranian databases including IranMedex and Irandoc by a community medicine specialist and a bachelor’s degree expert and eventually reviewed them. The titles and abstracts of the articles were evaluated, and those that were irrelevant according to the researchers’ opinions were excluded. The full texts of articles were assessed and for qualifying them, only the publication in accredited journals was considered. There were more than 300 articles related to Iraqi chemical war against Iranian people, in which 40 articles were related to mental health domain. There were no any ethical problems in processing this article.

## Results

Exposure to chemical weapons may lead to physical, mental, social, and economic damages and consequently leads to the decrease of QOL of the exposed people. In this study, we reviewed 68 articles that had been carried out on thousands of Iranian veterans and e summarized the results of these researches as follows:

Behdani et al. in their study conducted 200 veterans who were damaged physically and mentally, concluded that, 95% of the understudied cases suffered from different types of psychological disorders, and the frequency of these disorders among the chemically injured veterans was higher than the physically injured one (19). According to Zarghami et al. in Iraq-Iran War, the prevalence of these disorders was 85.50% among the 200 Iranian veterans and captured soldiers in Sari City, North of Iran (20).

Hashemian et al. in their study quoted Cardozo et al. in a national survey in Afghanistan, stated anxiety disorders (72%), depression (68%) and PTSD (42%) among the victims of war (21).

In Iran, Tavallaii assessed the mental health among 206 chemically victims in Sardasht (a city in Nort western Iran) by general health questionnaire (GHQ28 with 28 questions). The results showed that 95.10% of people had inappropriate mental health scores and the average of mental health score using the GHQ was 48.92 (22). In this test, the scores of 1-7 indicate mild impairment, 8-14 moderate impairment, and between 15 and 21 severe impairment of general health status. Therefore, the scores more than 22.50 indicate an abnormal status in this test. Hence, the above score in Tavallaii study indicated abnormal conditions in the habitants of the Sardasht. (22). Karami et al. in one descriptive-analytical study conducted on 100 veterans who suffered from MG effects also used GHQ. They determined that the average score of general health status among the understudied cases was 47.34. The average scores of the general health status among the understudied veterans were 12.80 in somatic symptoms, 13.63 in anxiety and insomnia, 10.73 in disability to perform daily activities and 10.16 in depression, respectively. The abnormal score was more than 6 for each component, which indicating health abnormalities in the participants (1). In Schnurr’s et al. study, most subjects (83%) had lower mental health than the same sample with similar ages in the general populations (23). The occurrence of depression and anxiety in chemical warfare in survivors were 7.20 and 14.60 times more frequently than the individuals with low-intensity warfare exposure (21). Tabatabai reported impaired consciousness (27%), low concentration (54%), emotional problems (98%), behavioral abnormalities (80%), thought processing disturbances (14%) and memory impairment (80%) three to 5 years after exposure among CIV (24). Neuro-psychological assessment of 1428 Iranian veterans 3-9 years after exposure to MG showed the following abnormalities: anxiety (15%), depression (46%), personality disorders (31%), seizures (6%) and psychosis (3%) (25). Vafaie, Seidy in one study conducted on 200 individuals (100 cases, 100 control) in Tabriz (a city in Northwestern Iran) showed that 100% of the chemically injured veterans who have been affected by 70% of damages had depression (26). Vafaie, Ghaderi in another study on 200 other veterans, described the rates of mental disorders as follows: major depression (23%), PTSD (19%), mania (13%), dysthymia (18%), generalized anxiety (4%), panic disorder (4%), schizophrenia (2%) and schizoaffective disorder (1.50%) (27).

PSTD, depression and anxiety are the important delayed complications discussed in the following parts.

***PTSD***

PTSD is one of the most common and important disorders in wars that can be caused as short as 1 week or as long as 30 years after trauma (28). This disorder is a syndrome that takes place in the face of a push factor causing severe damage. The exposure can be in the form of watching, participating, hearing or experiencing a war, torture, disaster, rape, assault, serious accident and fire. The victims review the traumatic events in their daily thoughts and dreams, and they avoid all of the things that would remind the stress and they have slow responses along with arousal state (26). Chemical weapon exposure as one of the most severe traumatic events can produce tensions, anxiety, loss of security and lead to chronic physical disabilities. The studies performed on chemically injured civilians showed many mental health problems (29).

PTSD affects not only the veterans’ QOL but also their families.' Repeated observations and clinical experiences showed that veterans’ families (wives and children) could suffer more psychological problems than the normal population (30, 31).

Ahmadi et al. in another study on 528 chemical warfare victims’ children of Sardasht in concluded that, the chemical warfare victims with PTSD may transfer the disorder to their children (31).

Clinical features of PTSD in our review were as follows: fear of painful re-experiencing of the event, try to avoid recalling it, feeling of repeating the experience, long-standing fatigue, feeling pressure in the head and neck, muscle tension and contraction, tremor, motionless like statues, excessive sweating, intermittent anorexia, vague abdominal discomfort, mild diarrhea, frequency (in urination), tachycardia, palpitations, dyspnea, chest pressure, dizziness, generalized fatigue, muscle weakness, feeling of guilt, depression, sleep disturbances, difficult concentration, irritability, outbursts of anger, persistent alertness, hyper reaction and attention deficit hyperactivity disorder in children (26). There are high levels of comorbidity between PTSD, anxiety and depression (21).

Hashemian et al. conducted a cross-sectional study on 153 habitants of three cities in the West Azarbaijan province of Iran in 2004. The characteristics of locations were as follows: Sardasht as a high intensity of both chemically and conventional warfare exposed town, Rabat as a non-chemically attached town and Oshnavieh, a town with lower intensity conventional warfare. The results are shown in table 1 (21).

Ahmadi et al. studied 150 cases of CIVs and 156 healthy men in 2002. They showed that in the veteran group 30 cases (20%) had severe stress disorders, 87 cases (58%) moderate and 3 mild stress disorders (32).

Trauma involves victims’ family members indirectly too. Ahmadi et al., in their study found that the frequency and severity of PTSD in CIVs’ spouses were significantly more common than the control group in Sardasht. It is named as secondary PTSD. Clinical signs and symptoms of PTSD and secondary PTSD are the same (30, 31). Also in another study, Ahmadi et al. studied 528 inhabitants of Sardasht City in two groups of cases (286 CIV) and controls (242 non-CIV). They evaluated secondary PTSD among the participants’ families. The results showed that the victims’ children are at greater risk for developing psychiatric and behavioral disorders special attention deficit hyperactivity disorder (31, 32). Also, Salimi et al. studied 52 CIV spouses and 52 mentally injured victims’ spouses in Mazandaran with the average age of 38 years. They showed that war may affect husbands’ partners, and they also may suffer from diverse psychological problems (33).

***Depression***

Depression is another common and important disorder among CIV. Vafaei and Seydi studied 100 injured veterans with 30-7% of injury and 100 healthy people. They were matched based on age and physical status. The frequency of depression among all the veterans was 71%, and this rate was 36% in the control group. The prevalence of depression among the chemically injured veterans was more than the non-chemically injured one, and this rate was 100% in CIVs with 70% of injury. Depression was related to the percentage of injury, and all of CIVs with higher than 50% of injuries were depressed (26). In Hashemian’s et al. study, the frequency of severe depression inhabitants of Sardasht, Rabat and Oshnavieh was 41%, 12% and 6%, respectively (21).

Suicide is one of the social pathological sign which is prevalent in depressive people. Tavallaii et al. in a retrospective study evaluated 1,463 veterans’ deaths. The cause of deaths in 70 (4.90%) of total cases was suicide. The frequencies of suicide based on the types were as follows: Hanging 19 (27.10%), drug intoxication 15 (21.40%), self-burning 14 (20%), suffocation 8 (11.50%), the use of gun 3 (4.30%), undefined 10 (14.30%) and others 1 (1.40%). The researchers stated that deaths due to suicide had occurred in lower age group (<40 years) (34).

***Other psychological disorders***

The frequencies of mental disorders in Iranian and non-Iranian studies were presented in table 2.

***QOL status in CIVs***

QOL is a concept that has different meanings for different people. This concept has dimensions of physical, mental, social and spiritual. Several factors may involve the QOL. Injuries induced by chemical weapons, especially MG, can alter people’s QOL and cause chronic and progressive disorders (56).

**Table 1:** Frequencies of anxiety and post-traumatic stress disorder among the inhabitants of Sardasht, Rabat and Oshnavieh in Western Iran

**City**	**Sardasht**	**Rabat**	**Oshnavieh**
**Kind of exposure**	High intensity of both conventional and chemically warfare %	Intense non-chemically war %	Lower intensity conventional warfare %
**Severe anxiety disorders**	65	26	18
**Lifetime PTSD** †	59	31	8
**Current PTSD** †	33	8	2

† Post-traumatic stress disorder

**Table 2:** Frequencies of mental disorders in performed studies in Iran and the other countries

**Disorder**	**Iranian studies results on chemical veterans % (reference)**	**Other studies results on general population % (reference)**
**Generalized anxiety**	4 (25) – 15 (23) – 18 to 26 (20) –65 (20)	57 (28) – 72 (20)
**PTSD** †	19 (25) – 41 (28) – 8 to 59 (20)	4 (34) – 7.8 (35) – PTSD in war trauma 20% (36) – 9 to 24 in Persian Gulf War veterans (37) – 18 to 37 (20) – 35 (22) – 42 (20)
**Phobia**	4 (25) – 62 (20)	Social phobia 3.7 (37) – 25 simple (36) – 49 Severe (36)
**Panic**	4 (25)	2.2 (38) – 2.4 million of American people (37)
**Sleep disorders**	93.6 (39)	20.1 (40)
**Anxiety insomnia**	59.2 (41)	29.9 (42) – 23.00% dissatisfied with their sleep, 29.90% had insomnia symptoms, 9.50% insomnia (42)
**Emotional problems**	98 (23)	Emotional/behavioral disorders are usually associated with communication disorders and they appear as: attention deficit/hyperactivity disorder, anxiety, and conduct disorder (43)
**Behavioral disorders**	80 (23)	communication disorders 6.3 (43) – intellectual disability 4.00% of CD cases – 3.70% with Autism (43)
**Personality disorders**	31 (21)	In America 9.1 (43) – 79.40% with at least one personality disorder (44)
**Paranoia symptoms**	61.5 (20)	18.6 (45)
**Obsessive-compulsive**	59 (1)	2 to 3 (46) –13(46)
**Psychosis**	3 (24)	3.48(47)
**Schizophrenia**	3.5 (25) Mania as a common symptom in schizophrenia13 (27)	14 (48) – 0.4 (49) – 0.87 (47) – schizoaffective disorder 0.32 (47) – schizophreniform disorder 0.07 (47) – 0.24 Bipolar (47)
**Depression (minor or major)**	6 to 41 (20) – 23 (25) – 46 (24) – 71 (25)	4.3 (50) – 5.3 (37) – 21 (48) – 35 (47) – 68 (20)
**Cognitive Impairment**	Automatic thoughts and well-being (51)	3.2 mild (52)
**Impaired awareness**	27 (23)	Different on the basis of diseases
**Low concentration**	54 (23)	Different on the basis of diseases
**Dysthymia**	18 (25)	1.6 (37) – 5 (53)
**Suicide**	5 (32)	8.9 suicidal feelings (54) – 0.6 actual suicide (55)

† Post-traumatic stress disorder

Direct effects of chemical injury on QOL are physical injuries, sleep disorders, lifestyle changes, drug addiction, interfering with everyday life, indifference and meaningless in life, having no plans for the future, depression, anger and invasion. In addition, indirect effects of chemical injury on QOL are decreased self-esteem, paranoia, nervousness and confusion and maladaptive life (10-12).

Berahmani et al. compared 232 injured people and 100 healthy people in Sardasht. They concluded that late complications of chemical injuries can cause physical, social and psychological limitations and decrease QOL. The results showed statistical relationships between QOL with employment, education level and severity of physical illness (12).

Safavi et al. studied QOL among 100 CIV’ spouses in the age range of 20-60 years and the mean age of 36.81. In physical aspects, only 19% of them had a good score, this score for the psychological and social aspects was zero (0) and 49%, respectively (56).

Mousavi et al. in their study about the QOL of 147 chemically ophthalmic injured victims reported the followings: The average age of injured people was 44.8 years, 98% were married, 28.60% had academic documents, and 28.60% had low level of education, too. Mostly (71.40%) were unemployed. Majority of them (72.10%) had a history of hospitalization. Both eyes were involved in 51.70%. The mean duration of exposure was 23.2 years. Mostly (54.40%) had sports activities. The frequencies of injuries were as follows: extremity injuries 57.30%, head injuries 27.40%, facial injuries 16.40% and psychological and psychiatric problems 32.90%. Mousavi et al. showed that QOL scores of CIV were significantly lower than the general population. They realized that the QOL of veterans who did not exercise were lower than others. There were relationships between poor mental health with more exposure time and low level of education (57).

Madarshahian compared QOL of 100 CIVs with 100 non-CIVs. She reported QOL problems in veterans as follows:

Irregular sleep pattern (55%), change in sleep duration (20%), nightmare (15%), tobacco use (60%), no rest (65%), no fun (70%), impairment in everyday life (65%), depression (40%), anger (40%), lack of desire to social activities (44%), low self-confidence (55%) and suspicion (45%). Madarshahian concluded that the CIVs show lower compliance than non-CIVs against challenges (11).

Chronic obstructive pulmonary disease (COPD) due to chemical exposure can decrease physical activities, family relationships, social contribution and daily performance and eventually lower QOL (58, 59).

Attaran et al. studied 43 male CIVs with COPD based on QOL in 2004. They revised St. George’s questionnaire according to social and cultural issues. They calculated the severity of disease via spirometry. They showed that the severity of COPD is negatively related to the QOL scores (58). St. George respiratory questionnaire is a standard tool for evaluating the QOL of patients with respiratory diseases. We can measure health status in persons with obstructive airway disease by this questionnaire (60). This instrument includes 76 items in three parts and assesses symptoms, activities and impacts. The symptom part evaluates the frequency and severity of respiratory symptoms. The activities part is related to the movements that are limited due to dyspnea, and the impact part is related to the social and mental functioning which is affected by respiratory disorders (58).

Aaron et al. showed that QOL scores in patients experiencing recurrent exacerbation of the disease had been decreased significantly, and they had more deaths (61).

Anxiety and mental tensions caused by chemicals injuries can activate some diseases like alopecia areata (62). The disease can consequently involve victims’ QOL.

***Sleep disorders***

Sleep disorders are common in CIVs (63, 40). Quality of sleep has an important role on daily activities and QOL in human beings (64). Many patients with PTSD have sleep disorders (63). During the Persian Gulf War in 1990, the following fatigue/sleep problems were observed in the soldiers:

Not feeling rested after sleep 42%, fatigue 36%, problems falling or staying asleep 33%, feeling unwell after exercise or exertion 17%, moderate or multiple fatigue symptoms 47% (65).

The frequency of depression is high in CIVs, and depressed people have more sleep problems than normal (66).

As abovementioned, chronic lung diseases are more common in CIV. COPD patients have nocturnal bronchospasm and wheezing (64). About 50% of such patients have poor sleep quality including delay in falling asleep, insomnia and night wakening. In other words, COPD patients have decreased arterial blood saturation and increased cholinergic tone and consequently nocturnal bronchospasm and sleep disturbances (67). Some common medications administered in lung diseases have negative effects on sleep too. For instance, theophylline induces poor sleep quality based on electroencephalogram studies (68). Tavallaii, Javadi vashki evaluated 78 CIV and 65 healthy controls matched for age and sex. They used Pittsburgh sleep quality questionnaire. This instrument evaluates the patients’ quality of sleep in the recent 4 weeks based on following 7 items:

1) delay in falling asleep, 2) duration of sleep, 3) adequacy of sleep, 4) waking up at nights, 5) use of hypnotics, 6) daily problems caused by poor sleep and 7) quality of sleep. The sensitivity and specificity of the questionnaire were 89.60% and 86.50%, respectively (69). Tavalaie et al. in another study found that 73 (93.60%) of CIVs and 39 (60.00%) of controls had poor sleep quality and the difference was statistically significant between groups (40).

## Discussion

In this study, we reviewed psychological disorders among chemically veterans after Iraq-Iran war that had been studied by researchers.

Overall, the general health scores earned by GHQ28, among CIVs were lower than the normal population in Iranian’s studies (1).

As it can be seen in table 2, the varieties of the studied disorders were:

Generalized anxiety, PTSD, phobia, panic, sleep disorders, anxiety insomnia, emotional problems, behavioral disorders, personality disorders, paranoia symptoms, obsessive-compulsive, psychosis, schizophrenia, depression, cognitive impairment, impaired awareness, low concentration, dysthymia, decide and decreased QOL.

The prevalence of generalized anxiety was 4-65% in this study versus 2.80-72.00% in the other studies, the rate of PTSD (8.00-59.00%) was more than the other studies.

In one study, 50% of American soldiers who had been exposed to MG in secret military experiments during World War II suffered from PTSD (69). Hashemian et al. quoted De Jong et al. showed that the prevalence of PTSD was estimated 37%, in Algeria 28% in Cambodia, 18% in Gaza and 16% in Ethiopia (21).

Dworkin et al. studied PTSD and self-perception and functioned (SPF) and stressful life events on 291 people of Halabcheh, a city in Iraq who had been exposed to chemical weapons by Iraqi forces. They showed that the frequency of PTSD and SPF was more common in the female gender, elderly and unemployed people than the others. These two mental disorders were significantly more common among people who had direct exposure to the event or they witnessed loss of their relatives (70). In general, psychiatric disorders among chemically veterans were higher than most other conditions.

At present, Iran is a unique country in the world in which tens of thousands of chemical warfare veterans live in this country (71). In addition to the studied disorders, a comprehensive national research should be done to improve the veteran’s QOL and in this study, the other psychiatric disorders should also be evaluated. 

We should find the coping resources in victims of chemical warfare. Coping is a process in which a person is actively involved during a period and she/he uses different strategies. Ebadi et al. in a qualitative study interviewed 20 males and females CIV (militaries and civilians) using semi-structured interviews and focus group discussions via a target based sampling method. They classified the adoption sources in four main categories including, religious factors, and feelings of patriotism, social support and attitude toward the diseases (72). Also, cognitive therapy can help disabled veterans to relieve their automatic thoughts (52).

## Conclusion

In most Iranian performed studies, the frequencies of PTSD, sleep disorders, emotional problems, behavioral disorders, personality disorders, paranoia symptoms, obsessive-compulsive disorders, depression, and mood disorders among the exposed chemical agents were higher than the other studies, which were performed in the unexposed population. Also, the QOL of veterans was lower than the other people in the community and the public health authorities and the non-governmental organizations should take more care of the veteran’s, especially their QOL. Finally, to deal with the problems we suggest strengthening their religious factors, patriotism, social support and positive attitude toward the diseases. 

## Notes

**Declaration of interest:** None.
